# Perceived benefits of cochlear implants by parents: expectations, decision-making process, and barriers to care

**DOI:** 10.1186/s42506-023-00132-w

**Published:** 2023-04-04

**Authors:** Ateya Megahed Ibrahim, Abdel-Hady El-Gilany, Eman Wardany Abdelaal Mohamed, Nesrine Saad Farrag

**Affiliations:** 1 Nursing Department, College of Applied Medical Sciences, Prince Sattam Bin Abdulaziz University, Alkharj, 11942 Saudi Arabia; 2 Family and Community Health Nursing, Faculty of Nursing, Port Said University, Port Said, Egypt; 3 Community Medicine Department, Faculty of Medicine, Mansoura University, Mansoura, Egypt; 4 Pediatric Nursing Department, Faculty of Nursing, Kafrelsheikh University, Kafr el-Sheikh, Egypt; 5 Community Medicine Department, Faculty of Medicine, Port Said University, Port Said, Egypt

**Keywords:** Cochlear implantation, Hearing disability, Health-related quality of life, Decision making, Access to health care

## Abstract

**Background:**

Identifying predictive factors of the cochlear implant outcomes in pediatric patients is critical in guiding tailored rehabilitation programs. The study aimed to assess cochlear implant outcomes, identify predictors, and highlight decision-making factors and barriers to quality care.

**Methods:**

This cross-sectional study included parents of children who received unilateral cochlear implants for bilateral severe- to- deep sensorineural hearing loss. Inclusion criteria were age ≤ 5 years and intelligence quotient (IQ) Scores ≥ 85, A pre-designed structured questionnaire was used to collect data from parents/guardians of the children attending follow-up. The Arabic validated Glasgow Children Benefit Inventory score was used to assess the health-related quality of life (QOL) after intervention.

**Results:**

The quality of life (QOL) score (outcome) after surgery was positive in all cases. Multivariate analysis showed that the site of operation (Bahtim hospital and Ain Shams Hospital [AOR(95% confidence interval CI), 5.7 (1.4–23), 5 (1.4–17.9), *p* = 0.015, 0.013, respectively]), education of the father (university/postgraduate [AOR (95% CI): 5 (1.4–17.9), *p* = 0.013]), the parents’ expectation that their child would be able to participate in regular classroom activities [AOR (95% CI): 8.9 (3.7–21.3), *p* < 0.001], and history of Attention deficit/hyperactivity disorder (ADHD), perinatal hypoxia, and low birth weight [AOR (95% CI): 2.5 (1.2–5.1), 3.7 (1.7–8.1), 4.7 (2.1–10.5), *p* = 0.013, 0.001, ≤ 0.001, respectively] are significant independent predictors of good outcome.

**Conclusion:**

All parents expressed a positive change in their child’s QOL. Almost all parents of children with cochlear implants face many barriers in obtaining quality healthcare services for their children. Parents, especially those with lower schooling, should receive good counselling to increase their confidence in their children’s capabilities and maximize benefits of regular follow-up. Improving the quality of healthcare centers is recommended.

## Introduction

Hearing loss (HL) among children is one of the most drastic disabling conditions that significantly impairs normal cognitive and language development. It prevents children from enjoying satisfactory social lives and can contribute to poor mental health and social isolation. Delayed management of the problem may result in degeneration of the central auditory system, leading to missed opportunities for education,, employment, and a lower quality of life [1, 2]. The prevalence of neonatal permanent sensorineural HL ranges from 0.1% to 0.6%, with an overall prevalence of 0.2% [3]. Early identification and management, including hearing augmentation within 6 months yields an optimal effect. Hearing screening programs in newborns enable the detection of hearing impairment in the first days after birth [4].

Many factors may lead to HL during different periods of the child’s life, including the prenatal factors (e.g., genetic factors, intrauterine infections), perinatal factors such as birth asphyxia, hyperbilirubinemia, and low-birth weight, or during childhood such as chronic suppurative otitis media, and meningitis) [5]. In some cases, the cause may be unknown accounting for 18.9% of cases [4].

The cochlear implant has significantly improved the treatment outcome for many children with severe to deep hearing disability. The criteria for candidacy and age of the surgery was driven by technological advancement [2, 6]. Parents of children with cochlear implant expect positive changes in language development, communication skills, academic progress, social life, and children’s future [7]. The expectations of parents play an important role in decision making to undergo through this surgery [8]. Expectations of parents are widely variable, depending on many factors, the most important are their level of education and awareness. Some of these expectations may be unrealistic and can cause of disappointment for some parents after experiencing the real outcome [9].

The likelihood of a better outcome for cochlear implant recipients increases when children and their parents have timely access to quality care. Barriers facing them to receive this quality care include individual factors like their socioeconomic level, family characteristics, and literacy level, or may be related to the health care system itself, including among others; regulations, equipment and the staff [10].

Although cochlear implant has become the standard treatment of cases of sever HL, the outcome of implant was poor in some cases. Clinical assessment alone is not enough to indicate positive changes in the quality of life (QOL) of these children. Additionally, there is no stipulated tool to measure the outcome of the implant. Some QOL tools have been developed and validated for cochlear implant users to include every positive change but these tools are still subjective [11].

Identifying the predictive factors of the outcome of cochlear implants is one of the most important goals as this is an invasive and expensive surgical procedure. This knowledge can help guide tailored rehabilitation programs to meet the expectations of clinicians, teachers, and parents. In addition to exploring the predictors of the outcome of cochlear implant, this research investigated the expectations of parents before surgery, the decision-making process, and the access to care as possible factors related to their judgment of the outcome of cochlear implantation. Although studies have approached parent’s experiences such as expectations and decision-making process, none of them have investigated whether they are associated with judgments of the outcome. This study aims to: 1) Assess the quality of life (QOL) of pediatric patients with bilateral moderate to severe HL after cochlear implantation (CI), 2) Outline the enabling and hindering factors of decision making and assess their association with the QOL, 3) Describe the barriers to healthcare and assess their association with the QOL, and 4) Identify the predictors of cochlear implant outcome in the included participants.

## Methods

### Study design

This study is a cross-sectional study with an analytical component.

### Setting and population

Cochlear implant surgery is performed at 19 centers/hospitals in Egypt under the umbrella of health insurance. The study used multistage cluster sampling. Using the random number function (RAND in Microsoft Excel), five centers were selected, namely Mansoura University Hospital, Sporting Hospital of Students in Alexandria, Bahtim Hospital, Wadi El-Nile Hospital, and Ain Shams Hospital.

Inclusion criteria: Parents of children aged ≤ 5 years with an IQ score ≥ 80, who received unilateral cochlear implant for bilateral moderate to deep sensorineural hearing loss, with at least one year of follow up after the surgery.

### Sample size

A previous study that used the Glasgow Children’s Benefit Inventory (GCBI) score for assessment of the outcome of cochlear implantation in children and found that the standard deviation (SD) was 24. Based on this SD, with a precision of ± 5 and confidence level of 95%, the calculated sample size was found to be 89 participants using the online sample size calculator (https://epitools.ausvet.com.au/onemean). Given the study's sampling strategy (multistage cluster sampling), a design effect of 2 was applied to adjust the sample size, resulting in a final sample size of 178. Two hundred children who met the inclusion requirements and attended the chosen locations between February and August 2021 were included in the study.

### Measurements/instruments

A pre-designed structured questionnaire was used to collect data from parents/guardians of the children who attended for follow-up. Data related to the child’s hearing history was obtained from the medical files of the patients. The questionnaire included the following sections:

#### Sociodemographic data

Sociodemographic data were collected using a validated Arabic questionnaire [12] for socioeconomic status. For the assessment of health literacy among the parents, we used the Single Item Health Literacy Screener (SILS). The SILS has one question: “How often do you ask someone for help to read the instructions and leaflets from a doctor or pharmacy?” The answers were recorded on a 5-point Likert scale: 5-never, 4-rarely, 3-sometimes, 2-often, or 1-always [13]. Adequate health literacy included parents who answered “never or rarely”, while inadequate health literacy included those answered “sometimes, often, or always” [14].

#### Child hearing history

This section included information on the timing and cause of deafness, use of auditory aids before implantation, duration of auditory deprivation, degree of hearing loss, presence of additional disabilities, and communication modality.

#### Clinical assessment data

This data was retrieved from the patients’ medical records. Data included assessment of the child’s hearing before surgery (e.g., IQ, degree of hearing loss), auditory performance score at the time of the interview, age at implantation, and any complications related to the surgery if applicable. The degrees of HL included in the study were dee HL (hearing threshold ≥ 91 dB), severe (hearing threshold: 71–90 dB), and moderate to severe (hearing threshold: 41–70) [15]. The auditory performance score [16] at the time of the interview was categorized as follows: no awareness of environmental sounds (0), awareness of environmental sounds (1), response to speech (2), identification of the environmental sounds (3), discrimination of common sounds without lip reading (4), understanding common phrases without lip reading (5, understanding conversation (6), and use telephone with known speaker (7).

#### Arabic validated Glasgow score

The Glasgow Children’s Benefit Inventory (GCBI) is a tool used to assess the health-related quality of life (QOL) after intervention in children who have undergone cochlear implant. The Arabic version of this tool was validated for assessment of the benefits of cochlear implant in children as reported by parents. The GCBI includes 24 items divided into 4 constructs; physical health (e.g., visits to the doctors, colds, need for medications), learning (e.g., absence from schools, leaning, concentration, distractibility, fun with friends), emotions (e.g., self-esteem, happiness, confidence, self-care), and vitality (e.g., overall life, things they do, progress, liveliness). The internal consistency of the tool was high (Cronbach’s α = 0.9). The GCBI uses a 5-point Likert scale ranging from 1 (much worse than before surgery to 5 much better than before surgery). The average of scores of these questions was deducted by 3, and then multiplied by 50 to obtain a benefit scale: -100 (i.e. maximal negative benefit) / 0 (i.e. no benefit) / + 100 (i.e. maximal positive benefit) [11].

#### Parental expectations

Parental Expectations regarding communication capabilities, social skills, academic achievement, and changes in future life of their Children, and knowledge of rehabilitation needs among parents. This questionnaire was adopted from Kumar et al. (2017) [7]. Answers were rated on a Likert scale from one (strongly disagree) to five (strongly agree). The questionnaire was translated into Arabic and back-translated into English to ensure linguistic validity and was reviewed for content validity by two independent public health experts. The score content validity index average of the items of this questionnaire (S-CVI) was 1. For statistical analysis, the answers were categorized into two groups: (1) (disagree/uncertain) (2) agree.

#### Decision making of parents

This section aims to explore how easy was the decision to have cochlear implant for their child. This section asked the participants about their involvement in decision making and the source of their first information about the surgery. This section used Likert scale questions ranging from 1 (strongly disagree) to 5 (strongly agree) to ask about the availability of information related to surgery, its complications, the need for follow up and if the decision to undergo surgery was a difficult one. For statistical analysis, the answers were categorized into 2 groups (1) agree and (2) (uncertain/disagree). Yes/no questions were then used to explore the reasons behind difficult decisions, and who supported the parents during decision-making [17].

#### Barriers to Care Questionnaire (BCQ)

Barriers to Care Questionnaire (BCQ): is a reliable tool used to assess sociobehavioral processes that negatively affect the patient's experience in the healthcare and reduce access to high quality care for children with special health needs. The BCQ is a multidimensional tool that includes five dimensions: expectations, marginalization, skills, knowledge, and pragmatics, with internal consistency reliability (alpha) of 0.95. Six questions from the original 39-item BCQ, were not included as they were not suitable for the Arabic culture. Each question has 5 possible answers (0 no problem, 1 a minor problem, 2 a problem, 3 big problems, 4 very big problem). It was translated into Arabic and back translated into English to ensure linguistic validity. Content and construct validity were checked by two independent public health experts [10]. The score content validity index average of the items of this section (S-CVI) was 1. For statistical analysis for each dimension of quality, categories of the answers were grouped into two categories (1) (no/minor problem) and (2) (a problem to very big problem).

### Data collection

Participants who met the inclusion criteria were interviewed at the selected centers using the study questionnaire. Data from the medical files of patients were accessed after approval from the healthcare centers and patients’ guardians. A pilot study was conducted with twenty parents at the Sporting Hospital of Students in Alexandria to ensure easy understanding and clarity of the tools.

### Ethical considerations

Nurses were informed about the objectives, purpose of the survey, expected benefits, types of information required, and publication of the findings before their participation. Only eligible parents who agreed to participate were included in the study. Informed written consent was obtained from all participants.

### Data analysis

Data was analyzed and tabulated using SPSS version 26. No missing data was found in the questionnaires completed by the researcher. Categorical data was presented as frequency and proportions. Nonparametric continuous data in Table 2 (e.g., age of the child, auditory performance score) were presented as categorical data based on their median. Patients with QOL score > median (41.7) were considered to have a good outcome compared to a poor outcome. Bivariate analysis was conducted to assess the different predictors of good outcome. Chi-square and Fisher’s Exact Tests were used wherever suitable. Variable found to be significant (*p* < 0.05) were entered into multiple binary logistic regressions to find the significant independent predictors of a good outcome.

## Results

**Fig. 1:**
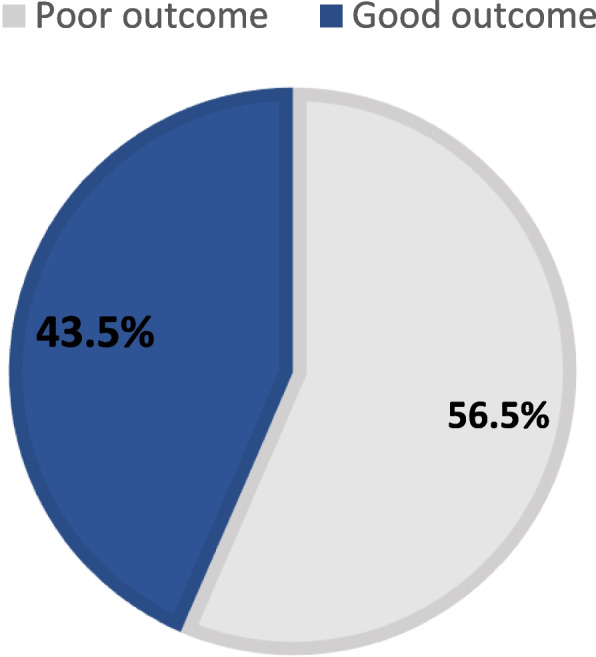
The quality of life after cochlear implant surgery score based on Glasgow Children’s Benefit Inventory (GCBI) questionnaire. Good outcome indicates score > median of 47.1

The study included 200 children with moderate to deep HL, recruited from five hospitals/centers in Egypt. The age of patients ranged from 2 to 5 years, with a mean age of 3 ± 0.9 years, 50% of them were males. Most of the children had severe HL (57.5%), and 42.5% of them had no family history of HL. In 60% of cases, parents did not know the cause of HL. All the children used pre-implantation hearing aids. The QOL score (outcome) after surgery was positive in all cases (i.e., they benefited from cochlear implant). It ranged from 18.8 to 62.5, with a median of 41.7 (the interquartile range of the median (IQR) was 33.3–47.9). Children with good outcome accounted for 43.5% of the sample. Figure 1 presents the QOL score results.

Table 1 demonstrates the relationship between parents' sociodemographic characteristics and the results of cochlear implantation. It shows that children treated at Ain Shams Hospital had considerably better outcomes compared to Mansoura University Hospital [Crude Odds Ratio (COR), 95% confidence interval (CI): 3.9, 1.4–10.6]. High levels of parental education (university/postgraduate) and maternal employment (administrative/professional) were linked to favorable outcomes [COR (95% CI): 5.4 (2.1–14.3), 2 (1.1–3.6)]. A statistically significant difference [COR (95% CI): 0.5 (0.2–0.9)] was found between parents with children aged 4–6 who reported a good result and parents with children aged 1–3 who reported a good outcome. Even though parents with higher health literacy reported better outcomes, there was no conclusive evidence linking the two variables. Parents' health literacy did not influence the result.

**Table 1:** The Association of sociodemographic characteristics of parents with the outcome of cochlear implant in children, Egypt, 2021

**Variable**	**Total**	**Good outcome** **N (%)**	**COR**^a^** (95% CI)**	***P* value**
Total	200	87 (43.5)		
**Education of mother**				
Illiterate/educated to preparatory level	58	25 (43.1)	1 (r)^b^	
Secondary/ middle institute	89	32 (36)	0.74 (0.37–1.45)	.756
University/ postgraduate	53	30 (56.6)	1.72 (0.81–3.65)	.155
**Education of father**				
Illiterate/educated to preparatory level	36	9 (25)	1 (r)	
Secondary/ middle institute	119	49 (41.2)	2.10 (0.91–4.85)	.079
University/ postgraduate	45	29 (64.4)	5.43 (2.06–14.34)	≤ .001
**Mother work**				
Not working/ manual	99	37 (37.4)	1 (r)	
Business/trade	15	3 (20)	0.41 (0.11–1.58)	.189
Administration/clerks/professional	86	47 (54.7)	2.02 (1.12–3.64)	.019
**Father work**				
Not working/ manual	75	30 (40)	1 (r)	
Business/trade	48	19 (39.6)	0.98 (0.46–2.06)	.963
Administration/clerks/professional	77	38 (49.4)	1.46 (0.77–2.77)	.246
**Hospitals**				
Mansoura University Hospital	25	7 (28)	1 (r)	
Sporting hospital of students, Alexandria	58	16 (27.6)	0.98 (0.34–2.78)	.969
Bahtim hospital	33	17 (51.5)	2.73 (0.90–8.27)	.072
Wadi El-Nile Hospital	24	11 (45.8)	2.17 (0.66–7.13)	.196
Ain Shams Hospital	60	36 (60)	3.85 (1.39–10.64)	.007
**Number of children**				
1–3	134	65 (48.5)	1 (r)	
4–6	66	22 (33.3)	0.53 (0.29–0.98)	.042
**Residence**				
Urban slums	74	30 (40.5)	1 (r)	
Rural	66	32 (48.5)	1.38 (0.71–2.69)	.345
Urban	60	25 (41.7)	1.05 (0.52–2.09)	.895
**Income**				
Insufficient	49	24 (49)	1 (r)	
Sufficient	76	53 (39.6)	0.68 (0.35–1.31)	.253
Can save money	58	10 (58.8)	1.48 (0.48–4.55)	.484
**Health literacy of parents**				
Inadequate	137	56 (40.9)	1 (r)	
Adequate	63	31 (49.2)	1.40 (0.77–2.55)	.270

*COR*^a^ Crude Odds ratio, (*r*)^b^ Reference

Table 2 presents the most important associations between various factors and cochlear implants. Most of the perinatal risk factors for HL, such as prenatal infections from the mother (*p* = 0.882), postpartum ICU hospitalization (*p* = 0.803), and neonatal jaundice (*p* = 0.705), were not significantly associated with the outcome. Operative problems including inner ear deformity (3.5%), improper electrode implantation (2.5%), and wound infection that required hospitalization (2.5%), were not significantly associated with the outcome (These are not shown in the tables). However, 3% of the children experienced negative outcomes and require re-implantation (*p* = 0.029).

**Table 2:** The Association of clinical/medical history of children with moderate/severe hearing loss with the outcome of cochlear implant, Egypt, 2021

**Variable**	**Total**	**Good outcome** **N (%)**	**COR**^a^** (95% CI)**	***P* value**
**Gender of the child**				
Boy	100	42 (42)	1 (r)^b^	
Girl	100	45 (45)	1.13 (0.65–1.98)	.669
**Age of the child (Y)**^c^				
≤ 3 Ys	144	67(46.5)	1 (r)	
> 3 Y	56	20 (35.7)	0.64 (0.33–1.21)	.166
**Age at the time of operation (years)**^c^				
≤ 2 Y	143	68 (47.6)	1 (r)	
> 2 Y	57	19 (33.3)	0.55 (0.29–1.04)	.067
**Significant Perinatal period conditions**				
**Low birth weight**				
No	117	43 (36.8)	1 (r)	
Yes	83	44 (53)	1.94 (1.09–3.44)	.022
**Asphyxia**				
No	79	24 (30.4)	1 (r)	
Yes	121	63 (52.1)	2.49 (1.37–4.53)	.002
**Past /present history of comorbidities**				
**ADHD**				
No	102	37 (36.3)	1 (r)	
Yes	98	50 (51)	1.83 (1.04–3.22)	.035
**Other disabilities**				
No	148	59 (39.9)	1 (r)	
Yes	52	28 (53.8)	1.76 (0.93–3.32)	.08
**Other diseases**				
No	127	52 (40.9)	1 (r)	
Yes	73	35 (47.9)	1.33 (0.74–2.37)	.336
**Preoperative factors**				
**Age of the child at onset of hearing loss**^c^				
≤ 8 months	101	52 (51.5)	1 (r)	
> 8 months	99	35 (35.4)	0.52 (0.29–0.90)	.021
**Degree of HL**				
Deep (≥ 91 dB)	27	8 (29.6)	1 (r)	
Sever (71–90 dB)	115	49 (42.6)	1.76 (0.71–4.35)	.216
Moderate to severe (41–70)	58	30 (51.7)	2.55 (0.96–6.74)	.056
**Family history of HL**				
No	85	42 (49.4)	1 (r)	
First degree relative	49	21 (42.9)	0.77 (0.37–1.56)	.464
Others	66	24 (36.4)	0.59 (0.30–1.13)	.109
**Parents know cause of deafness**				
No	120	51 (42.5)	1 (r)	
Yes	80	36 (45)	1.11 (0.63–1.96)	.727
**IQ before operation**				
80–90	35	15 (42.9)	1 (r)	
90–100	57	25 (43.9)	1.04 (0.45–2.44)	.925
100–110	66	29 (43.9)	1.05 (0.45–2.39)	.917
110–120	42	18 (42.9)	1 (0.40–2.48)	1
**Auditory performance score**^cd^				
≤ 3	109	48 (44)	1 (r)	
> 3	91	39 (42.9)	0.95 (0.54–1.67)	.867
**Post-operative factors**				
**Need for re-implantation**				
No	194	87 (44.8)		
Yes	6	0	Not applicable	
**Regular post-operative vocal/verbal training**				
No	21	4 (19)	1 (r)	
Yes	179	83 (46.4)	3.67 (1.18–11.35)	.037
**The language used to communicate**				
spoken	43	24 (55.8)	1 (r)	
Sign	47	18 (38.3)	0.54 (0.26–1.11)	.096
both	110	45 (40.9)	0.57 (0.31–1.01)	.096
**Duration of using the device daily (hours)**^c^				
≤ 10 h	123	60 (48.8)	1 (r)	
> 10 h	77	27 (35.1)	0.6 (0.3–1)	.057

*COR*^a^ Crude Odds ratio, (*r*)^b^ Reference, ^c^ Cut off points for these variables are based on Median, ^d^ grading of the auditory performance score: no awareness of environmental sounds (0), awareness of environmental sounds (1), response to speech (2), identification of the environmental sounds (3). Discrimination of common sounds without lip reading (4), understanding common phrases without lip reading (5), understanding conversation (6), Use telephone with known speaker (7)

**Fig. 2:**
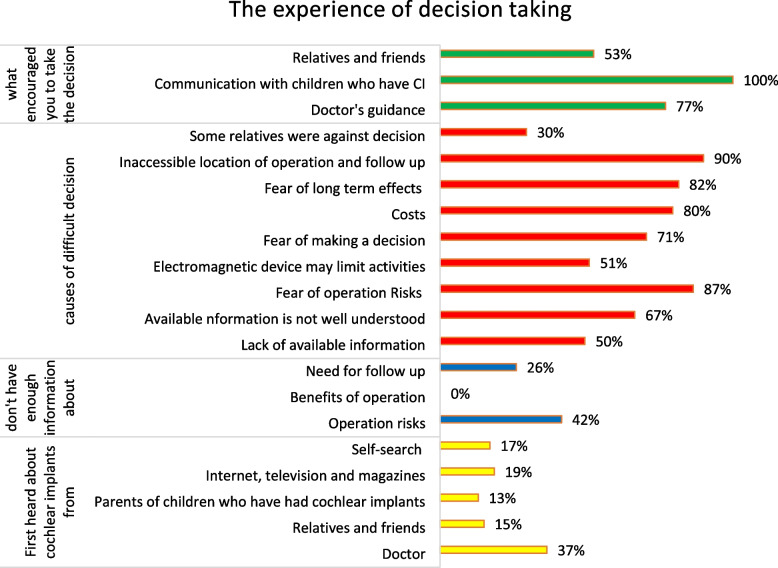
The experience of decision making by parents of children with cochlear implants. CI: cochlear implant

**Fig. 3:**
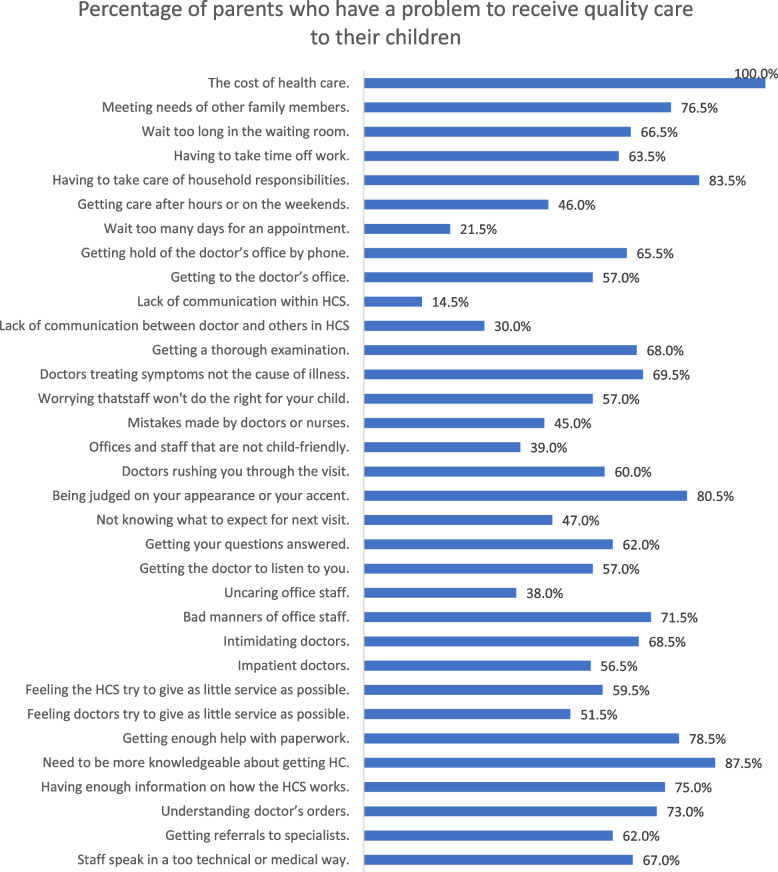
Barriers expressed by parents in accessing quality healthcare for their children with cochlear implants within the healthcare system (HCS)

Figure 2 presents the details of the decision-making process. Decision-making was not significantly associated with the outcome (Table 3). Figure 3 shows the barriers to quality care.

**Table 3:** The Association of expectations of parents before cochlear implantation, decision making process, barriers to quality care, and the outcome of children with cochlear implant, Egypt 2021

Variable	Total	Good outcome N (%)	COR^a^ (95% CI)	*p* value
**Significant expectations before cochlear implantation**				
**Child will be able to participate in regular classroom activities**				
Uncertain/disagree	82	20 (23.3)	1 (r)^b^	
Agree	114	67 (58.8)	4.70 (2.52–8.78)	≤ .001
**Parents will have to put a lot of effort into the rehabilitation process**				
Uncertain	20	14 (70)	1 (r)	
Agree	180	73 (40.6)	0.29 (0.11–0.79)	.012
**Decision making**				
**Making decision was difficult**				
Disagree	19	10 (52.6)	1 (r)	
Agree/ uncertain	184	77 (42.5)	0.67 (0.25–1.72)	.399
**Hesitated to decide**				
Disagree	89	34 (38.2)	1 (r)	
Agree/ uncertain	111	53 (47.7)	1.47 (0.84–2.61)	.176
**Significant barriers to care**				
**Having to take time off work**				
No/simple problem	73	40 (54.8)	1 (r)	
Problem/very big	127	47 (37)	0.49 (0.27–0.87)	.015
**Lack of communication between various parts of the health care system**				
No/simple problem	171	81 (47.4)	1 (r)	
Problem/very big	29	6 (20.7)	0.29 (0.11–0.75)	.007
**Judged by appearance, ancestry, or accent**				
No/simple problem	39	23 (59)	1 (r)	
Problem/very big	161	64 (39.8)	0.46 (0.23–0.94)	.03
**Not knowing what to expect from one visit to the next**				
No/simple problem	106	55 (51.9)	1 (r)	
Problem/very big	94	32 (34)	0.47 (0.27–0.84)	.011

*COR*^a^ Crude Odds ratio, (*r*)^b^ Reference

Table 3 presents the significant factors associated with expectations and barriers to quality care. Barriers found to be significantly associated with the outcome are shown in Table 3. Other barriers that were found to be insignificant include parental skills (e.g., understanding physicians’ orders, obtaining help to fill in forms, having sufficient information about the healthcare system), marginalization (e.g. feeling that physicians give them suboptimal service, impatient physicians, and carless healthcare staff), expectations (e.g. offices and staff being not suitable for children, and absence of communication between healthcare staff), pragmatism (long waiting time, the ease of making appointments, and the cost of healthcare).

Table 4 shows the results of multivariate analysis of the predictors of a good outcome. It indicates that the hospital/center where the operation was performed (Bahtim hospital and Ain Shams Hospital [Adjusted Odds ratio (AOR) (95% CI), 5.7 (1.4–23), 5 (1.4–17.9), *p* = 0.015, 0.013, respectively]), high education level of the father (university/postgraduate [AOR (95% CI): 5 (1.4–17.9), *p* = 0.013], the parents’ expectation that their child would be able to participate in regular classroom activities [AOR (95% CI): 8.9 (3.7–21.3), *p* < 0.001], the presence of AHDS [AOR (95% CI): 2.5 (1.2–5.1), *p* = 0.013], positive history of perinatal hypoxia [AOR (95% CI): 3.7 (1.7–8.1), *p* = 0.001], positive history of low birth weight [AOR (95% CI): 4.7 (2.1–10.5), *p* < 0.001] were significant independent predictors of a good outcome.

**Table 4:** Multivariate Logistic Regression of predictors of good outcome of cochlear implantation among children with moderate/severe hearing loss, Egypt, 2021

**Predictors**	β	*p*	AOR^a^ (95% CI^b^)
**Hospitals (site of the operation)**			
Mansoura University Hospital			1 (r)^c^
Sporting hospital of students, Alexandria	0.40	.543	1.50 (0.40–5.60)
Bahtim hospital	1.73	.015	5.68 (1.40–23.00)
Wadi El-Nile Hospital	0.76	.300	2.14 (0.51–9.06)
Ain Shams Hospital	1.62	.013	5.04 (1.41–17.95)
**Education of the father**			
Illiterate/educated to preparatory level			1 (r)
Secondary/ middle institute	0.88	> .999	2.42 (0.84- 6.93)
University/ postgraduate	1.883	.003	6.57 (1.92–22.42)
**Parents expected Child would be able to participate in regular classroom activities**			
Uncertain/disagree			1 (r)
Agree	2.18	< .001	8.92 (3.74–21.27)
**AHDS**			
No			1 (r)
Yes	0.91	.013	2.48 (1.21–5.08)
**Perinatal asphyxia**			
No			1 (r)
Yes	1.31	.001	3.71 (1.69–8.11)
**Low birth weight**			
No			1 (r)
Yes	1.56	< .001	4.73 (2.14–10.46)
**Constant**	-5.57		
**Model Chi-Square**	79.89, < .001		
**Percent correctly predicted**	76%		

*AOR*^a^ Adjusted Odds Ratio, CI^b^ Confidence interval, (*r*)^c^ Reference group

## Discussion

The current study aimed to assess the change in quality of life of children with severe HL after cochlear implant surgery and to identify its predictors. The results showed that all parents expressed a positive change in their child’s QOL. In pediatric population, parent’s assessment of QoL is a reliable indicator of the QoL experienced by children [18]. Parents play a critical role in evaluating their child's well-being and the outcomes of any therapeutic intervention. It is now well-established that cochlear implants greatly improve the QOL of most patients. This consensus was reported in several self-reported and parent-reported questionnaires [17–19]. Interestingly, QoL may be comparable to that of children with normal hearing, without significant differences as found in Alnuhayer et al.’s study [20]. This study found no significant difference in any QOL domain as reported by their parents, between normal children (2–7 Y) and those with cochlear implant [20].

The results showed that father’s education was a significant independent predictor of the outcome. Parents with high education reported good outcome nearly six times more often than those with primary or no education as shown in the regression analysis. In addition, mothers’ employment status and the number of children in the family were significantly associated with the outcome. These associations may be related to early detection and diagnosis of the problem. Also, these factors may indicate a higher socioeconomic status, and better literacy, which are predictors of medications adherence and follow up care [13]. The commitment to regular post-operative vocal/speech training was significantly associated with the outcome as shown in the results. Good outcomes were reported more often among parents with adequate literacy (49.2%) compared to those with inadequate literacy (40.9%).

Parental factors are the most common modifiable barriers to early implantation [21]. Additionally, one study in Brazil found a significant correlation between mother’s level of education, but not father’s, and some domains of QoL after cochlear implantation [22]. In contrast, the level of education of both mother and father was not associated with the QOL in another study of Saudi children [20]. It is optimistic to find that the outcome in our study is not related to the income of the family which is consistent with other studies [18, 23]. The level of parents’ education and their purchasing power should not be barriers to achieving better QoL after cochlear implantation.

The hospital or center where cochlear implant was performed, was found to be a significant independent predictor of the outcome. After controlling for other variables, multivariate analysis showed that a good outcome was reported in two hospitals (Bahtim hospital and Ain Shams Hospital) five times more often than in Mansoura University hospital. The present study highlights the importance of conducting diagnostic, therapeutic, and rehabilitation processes in specialized centers or hospitals with extensive, prolonged, and proven experience as also reported by Busi et al. [24].

Several studies have reported that early implantation (before 24 months) was associated with better outcome, especially when evaluated in older ages such as after the age of 12 Years in some studies. which suggests a longer duration of device use [18, 21, 24]. However, in our study, analysis of the clinical history of the children indicates that neither the age at surgery nor the age of the child at evaluation was associated with the outcome. This may be because our study included a homogenous group of participants aged ≤ 5 years, with median age of implantation at 24 months. Our results are consistent with results reported by Alnuhayer et al. [20]. Similarly, except the communication domain, which was higher in children with early implantation (< 24 months) [9], none of the QOL domains were associated with the age of implantation.

Our study found that among the causes and risk factors of hearing loss (HL), children who were low birthweight (LBW) or had asphyxia were more likely to have good outcome (AOR (95% CI): 4.7 (2.1–10.5) 3.7 (1.7–8.1), respectively). Additionally, among comorbid conditions, ADHD was found to be the only comorbidity associated with positive outcome. Children with ADHD had a higher likelihood of good outcomes [AOR (95% CI): 2.5 (1.2–5.1)]. Research suggests that the different etiologies of HL may predict the child's listening and language development differently after cochlear implant. Some etiologies of HL may also cause additional difficulties or comorbid congenital malformations. Some studies found that children who were deafened by meningitis or cytomegalovirus as well as those with auditory neuropathy spectrum disorder had a higher number of additional comorbidities such as epilepsy and autism, while some congenital causes had almost no additional difficulties [20, 25]. Our findings align with Cejas et al.’s report, which indicated that comparisons of outcomes of cochlear implant across associated disabilities showed that children with little to no cognitive impairment such as ADHD, had better outcomes than those with greater deficits in intellectual functioning, such as autism and CHARGE syndrome [26].

We hypothesized that parental expectations prior to cochlear implant are related to QoL reported by parents after the procedure, and our study confirmed this hypothesis. Parents who anticipated that their children would participate in regular classroom activities had a nearly five-fold greater chance of reporting a positive outcome even after controlling for other variables. This finding is interesting and may be explained by parent's intent to help their children participate in regular schools and to support them to a achieve this goal. Of note that, in a secondary analysis of our data, we found a highly significant association between this expectation and regular vocal/verbal training.

The decision to undergo cochlear implantation was reported to be the most challenging period for the parents in their journey with the procedure [9], and hesitation may serve as a barrier against early implantation [21]. In our study, we hypothesized a significant association between decision making and the outcome. However, our results indicated otherwise. To the best of our knowledge, this point has not been explored by previous studies. Several factors made the decision difficult for many parents, including the inaccessible location of operation and follow up (90%), concerns about operation risks (87%), potential long-term effects (82%), and costs (80%). Moreover, our study revealed that 67% of parents had difficulty understanding the available information with the most commonly deficient information being related to the health risks associated with the operation. Therefore, counselling of parents during the preoperative period is crucial for relieving parental anxiety during decision making and preventing unrealistic expectations. Parents first learned about cochlear implantation primarily through doctors, but the key factor that encouraged parents to take the decision was communication with children who had already undergone cochlear implantation (100%), followed by guidance from doctors (77%).

Receiving quality care was challenging for most of parents, because of a lot of barriers. The most prevalent barrier was cost which was a problem for all parents. Additionally, needing to be more knowledgeable about obtaining health care was among the most prevalent barriers (87.5%). Our results showed that although some of the barriers were significantly associated with poor outcomes, as hypothesized prior to the study, they were no longer significant after controlling for other variables, these barriers could not only decrease parental compliance but also delay the ideal timing of implantation. Armstrong et al. reported that difficulties in navigating the system, non-compliance with candidacy evaluation appointments, and misunderstanding of candidacy process are parental factors that delay the operation [21].

### Limitations of the study

As a cross-sectional study, our research was subject to biases that commonly affect this type of study, such as recall bias. Additionally, as parents had already observed the outcome, their expectations prior to surgery may have been biased at the time of evaluation. The evaluation of the outcome in our study was entirely subjective. However, this subjective evaluation was a study objective, as we aimed to investigate how the surgery changed parents' lives.

## Conclusion

Our results emphasize several independent predictors of a favorable outcome after cochlear implantation. Some of these predictors are risk factors for deafness including low birthweight, birth asphyxia, and AHDS. Other factors included the hospital/ center where the surgery was performed, the level of education of the father, and parents’ expectations that the child will participate in regular classroom activities.

Our results highlight the fact that almost all parents of children with cochlear implants face many barriers in obtaining quality healthcare services for their children. Some of these barriers are related to their judgment on the outcome of cochlear implantation. Decision making is a challenging period of parents’ life that requires support to make easier. The most encouraging factor is having contact with other children who had undergone cochlear implantation.

Future prospective research is necessary to further explore the association between parents’ expectations and the outcome of cochlear implantation. Good counselling and educational messages should be provided to parents especially those with lower levels of education. Also, encouraging parents to maximize the benefits of regular follow up and increase confidence in their children’s abilities is essential. Choosing a high-quality center with sufficient experience is recommended for achieving a positive outcome.

Policy makers should consider unofficial or charity groups of friends or communities of deaf children to support parents especially whose child has just been diagnosed as deaf. Directing these parents to these groups could help doctors in providing the necessary counselling of these parents.

Our findings can help the interdisciplinary team of implantation improve counselling for parents, starting from the decision-making period and continuing throughout the course of rehabilitation with customized plans for messages and support for children and their families. This could take into consideration their different socioeconomic and literacy levels, ensuring that families have equal opportunities to full access to understandable information and to maximize the benefits of follow-up.

## Data Availability

The data underlying this article is available in [Mendeley Data repository, https://doi.org/10.17632/jx4wksk4gp.1], and will be published once the paper is accepted at this link: https://doi.org/10.17632/jx4wksk4gp.1.

## Abbreviations

HL: Hearing loss
QOL: Quality of life
IQ: Intelligence quotient
SILS: Single Item health literacy Screener
